# Hierarchical Nanocomposites Electrospun Carbon NanoFibers/Carbon Nanotubes as a Structural Element of Potentiometric Sensors

**DOI:** 10.3390/ma15144803

**Published:** 2022-07-09

**Authors:** Barbara Niemiec, Marcel Zambrzycki, Robert Piech, Cecylia Wardak, Beata Paczosa-Bator

**Affiliations:** 1 Faculty of Materials Science and Ceramics, AGH University of Science and Technology, Mickiewicza 30, 30-059 Krakow, Poland; bniemiec@agh.edu.pl (B.N.); zambrzycki@agh.edu.pl (M.Z.); rpiech@agh.edu.pl (R.P.); 2 Department of Analytical Chemistry, Faculty of Chemistry, Institute of Chemical Sciences, Maria Curie-Sklodowska University in Lublin, Maria Curie-Sklodowska Sq. 3, 20-031 Lublin, Poland; cecylia.wardak@poczta.umcs.lublin.pl

**Keywords:** hierarchical nanocomposites, carbon nanotubes/carbon nanofibers, potentiometric sensor, potassium determination, hydrophobic material

## Abstract

This work proposes new carbon materials for intermediate layers in solid-contact electrodes sensitive for potassium ions. The group of tested materials includes electrospun carbon nanofibers, electrospun carbon nanofibers with incorporated cobalt nanoparticles and hierarchical nanocomposites composed of carbon nanotubes deposited on nanofibers with different metal nanoparticles (cobalt or nickel) and nanotube density (high or low). Materials were characterized using scanning electron microscopy and contact angle microscopy. Electrical parameters of ready-to-use electrodes were characterized using chronopotentiometry and electrochemical impedance spectroscopy. The best results were obtained for potassium electrodes with carbon nanofibers with nickel-cobalt nanoparticles and high density of nanotubes layer: the highest capacity value (330 µF), the lowest detection limit (10^−6.3^ M), the widest linear range (10^−6^–10^−1^) and the best reproducibility of normal potential (0.9 mV). On the other hand the best potential reversibility, the lowest potential drift (20 μV·h^−1^) in the long-term test and the best hydrophobicity (contact angle 168°) were obtained for electrode with carbon nanofibers with cobalt nanoparticles and high density of carbon nanotubes. The proposed electrodes can be used successfully in potassium analysis of real samples as shown in the example of tomato juices.

## 1. Introduction

In the field of potentiometry, research is largely focused on the development and modification of ion-selective electrodes. The simplest structure of an ion-selective electrode (ISE), called a coated disc electrode, is a conductive substrate directly covered by an ion-selective membrane (ISM) [1]. However, such a simple design is quite unreliable due to the following problems: (i) instability of the potential; (ii) poor reproducibility caused by the direct connection of materials of different types of conductivity. The charge-transfer process at the interface between electronically conducting substrates and ISM is disturbed, which results in deterioration of the potentiometric response. The easiest way to eliminate these problems is to place an additional electroactive material between the substrate and the ion-selective membrane. Such structured electrodes are called solid-contact electrodes. However, many materials have been tested as intermediate layers, new solutions are still sought [2,3].

Nanomaterials are one of the groups of materials used as intermediate layers [4]. The physical and chemical properties of nanomaterials make them very useful in the construction of potentiometric sensors. The large contact area with the ion-selective membrane provides a high electric charge capacity. This allows to achieve high stability of the sensor potential. Additionally the high hydrophobicity eliminates the problem of the formation of a water layer at the membrane—solid-contact interface. These features are one of the main requirements for materials used as intermediate layers [3,4]. Among these nanomaterials, carbon based materials such as single [5] and multiwall [6] nanotubes, graphene [7], chemically reduced graphene oxide [8] and carbon black [9] have been successfully applied.

Hierarchical carbon nanocomposites are a new group of carbon nanomaterials. They are characterized by a strictly designed architecture and a unique structure. Branched carbon nanofibers are typical examples of these materials [10]. They are a composite of nanofibers and carbon nanotubes (CNF/CNT). Carbon nanofibers are the core and nanotubes are attached perpendicularly to the surface. This structure determines: (i) the large surface/volume ratio, (ii) the increase in the number of active sites for adsorption and catalysis, and (iii) the good conductivity of the material [11,12,13]. Due to these properties, carbon nanocomposites are prospective in terms of use as intermediate layers in solid-contact electrodes.

The basic method for the synthesis of CNF/CNT nanocomposites was proposed by Hou and Reneker [14] and consists of two steps. The first stage is the electrospinning of polymer nanofibers with a precursor of metallic nanoparticles (metal acetylacetonates). Subsequently is thermally treated and combined with the gas-phase synthesis of nanotubes. During the thermal treatment, the nanofibers carbonize and the metal precursor decomposes, leaving evenly distributed metal nanoparticles that catalyze the growth of the nanotubes. The used catalyst precursor also influences the properties of the synthesized nanocomposite [11,15].

This paper proposes the use of a number of materials as an intermediate layer in ion-selective electrodes:

- electrospun carbon nanofibers (eCNF),
- eCNF with Co nanoparticles deposited on the surface (eCNF-Co),
- hierarchical nanocomposite with Co nanoparticles as a CNT growth catalyst (eCNF/CNT[HD]-Co),
- two hierarchical nanocomposites eCNF/CNT with NiCo nanoparticles as growth catalysts differing in the surface density of CNT:
  - with high density eCNF/CNT[HD]-NiCo
  - with low density eCNF/CNT[LD]-NiCo.

Carbon nanofibers have already been used as solid contact for the determination of the multichiral drug moxifloxacin [16]. The other proposed materials are new in this field. This work presents the first attempt to use them as intermediate layers in ion-selective electrodes.

The aim of this work is to apply the proposed materials as an intermediate layer in ion-selective electrodes. The goal is to obtain layers with a large specific surface area. The resulting hydrophobicity of the material prevents the formation of a water layer. The large surface/volume ratio of the material also translates into a number of active sites, which allows for obtaining sensors with a high electric charge capacity and thus a stable response.

## 2. Materials and Methods

### 2.1. Chemicals

Five different nanomaterials were tested as an intermediate layer in the construction of solid-contact electrodes. Dispersions of material in DMF (dimethylformamide) contained 5 mg/mL of the following materials: eCNF, eCNF-Co, eCNF/CNT[HD]-Co, eCNF/CNT[HD]-NiCo, and eCNF/CNT[LD]-NiCo. The materials were prepared according to the method described by Zambrzycki [11]. A two-stage process includes (i) the preparation and heat treatment of electrospun carbon nanofibers, and (ii) their modification by CNT synthesis on the nanofiber surface by catalytic chemical vapor deposition (CCVD). The synthesis of hierarchical nanocomposites was previously fully described [15,17]. The synthesis of eCNF includes only first-step electrospinning without adding a precursor for growth of nanotubes and thermal treatment. The synthesis eCNF-Co also includes only these steps, but to the precursor solution a 3% of Co(Acac)_2_ (99%, ACROS Organics^TM^, Geel, Belgium) was added. In the synthesis of the rest of the materials, the mixture contains 3% of Co(Acac)_2_ or 1.5% of Ni(Acac)_2_ (>98%, ACROS Organics^TM^, Belgium) and 1.5% of Co(Acac)_2_. Different flows of carrier gas were used in the synthesis of nanotubes by the CCVD method. As a result, materials with different density of carbon nanotubes on the nanofiber surface were obtain. For material with a low density of nanotubes (eCNF/CNT[LD]-NiCo), a flow of C_2_H_2_/N_2_:Q = 15/450 mL min^−1^ was used, and for materials with a high density of nanotubes (eCNF/CNT[HD]-Co and eCNF/CNT[HD]-NiCo), a flow of C_2_H_2_/N_2_:Q = 28/450 mL min^−1^. One synthesis yields 3–4 mg of material.

The membrane components: potassium ionophore I (Valinomycin), lipophilic salt-potassium tetrakis(4-chlorophenyl)borate (KTpClPB), 2-nitrophenyl octyl ether (o-NPOE) and poly(vinyl chloride) (PCV) and tetrahydrfuran (THF) as solvent were selectophore reagents purchased from Sigma-Aldrich (Saint Louis, MI, USA).

Potentiometric, chronopotentiometric and electrochemical impedance spectroscopy measurements were performed with potassium chloride solutions (analytical reagent grade, POCH, Gliwice, Poland) and sodium chloride solutions (analytical reagent grade, Sigma-Aldrich, Saint Louis, USA) prepared from 1 M standard solution. The aqueous solutions were prepared with distilled and deionized water.

### 2.2. Electrode Preparation

The drop casting method was selected for the preparation of the intermediate layer and the ion-selective membrane. This method allows to obtain the all-solid-state electrode easily and quickly. Before dropping the layer solution, glassy carbon (GC) disc electrodes (Mineral, Warsaw, Poland) were polished with alumina powder and ultrasonically cleaned with water and methanol. The layer solutions were dispersed just before application to the electrode surface using an ultrasonic homogenizer for 5 min. 

15 μL of layer solution was dropped to the top of a clean and dry electrode and in this way were prepared three electrodes each type. Then the electrodes with layers were left to dry by evaporating at room temperature. After evaporating the solvent, the electrodes were covered with 70 μL of K^+^ ion selective membrane solution containing potassium ionophore I 1.10% (*w*/*w*), KTpClPB 0.25% (*w*/*w*), o-NPOE 65.65% (*w*/*w*), PVC 33.00% (*w*/*w*). Components of 0.125 g total weight were dissolved in 1 mL of THF. All electrodes were left at room temperature to evaporate the solvent. After this time, the electrodes were placed in 0.01 M KCl to be conditioned. A group of electrodes called coated disc electrodes was also prepared as a control group. This group was prepared by directly covering the electrode surface with an ion-selective membrane.

### 2.3. Apparatus and Methods

The morphology of the materials tested as intermediate layers was characterized after synthesis using a scanning electron microscope, model LEO 1530, from LEO Electron Microscopy Ltd., New York, NY, USA.

The wettability of each tested layer was determined by measuring the wetting angle using a Theta Lite contact angle microscope (Biolin Scientific, Gothenburg, Sweden) with One Attension software. The water was dropped onto the tested layer that covered the GC disc.

Potentiometric measurements were made using a 16-channel EMF meter (Lawson Labs, Inc., Malvern, PA, USA). Ag/AgCl electrode with 3 M KCl in a bridge cell (6.0733.100 Ω Metrohm, Herisau, Switzerland) or Ag/AgCl/3 M KCl with 1M LioAc in a bridge cell (6.0729.100 Ω Metrohm, Switzerland) were used as the reference electrode and a platinum rod as the auxiliary electrode.

Electrochemical impedance spectroscopy and chronopotentiometric measurements were performed using the Autolab General Purpose Electrochemical System (AUT302N.FRA-2-AUTOLAB, Eco Chemie, Utrecht, The Netherlands) that cooperated with the NOVA 2.1.4 software. Measurements were carried out in a three-electrode system with Ag/AgCl/3 M KCl electrode (6.0733.100 Ω Metrohm, Switzerland) as reference electrode, a carbon glass rod as auxiliary and an indicator electrode with a particular layer. All measurements were carried out in 10^−2^ M KCl solution.

Electrochemical impedance spectroscopy allowed to determine the electric capacity of the electrodes. Measurements were made in the frequency range of 100–0.01 Hz with an amplitude of 10 mV superimposed on the open circuit potential (OCP). The electric capacity was determined from the dependence C = 1/(2πƒZ″) using the value of the imaginary part of an impedance at a frequency of 0.01 Hz.

The method of chronopotentiometry allowed to determine the electrical parameters of the tested electrodes. According to the method proposed by Bobacka [18] the dependence of the potential on time during the forced current flow through the system allows to determine parameters such as potential drift, resistance and electric capacity. The drift of the potential is defined as the derivative of the potential over time (ΔE_dc_/Δt) and on its basis the electric capacity can be determined from the dependence C = I(Δt/ΔE_dc_). The resistance is determined from the equation R = E/I, where E is the potential jump when the direction of the current flow changes. The current value proposed in the method (±1 nA) was not sufficient to obtain interpretable chronopotentiograms for electrodes with carbon layers, which results from their high electrical capacity. A current of ±10 nA was used to obtain a change in the potentiometric response for these electrodes. The response was recorded for 60 s for each direction of current flow.

## 3. Results and Discussion

### 3.1. Microstructure Analysis by Scanning Electron Microscopy 

**Figure 1:**
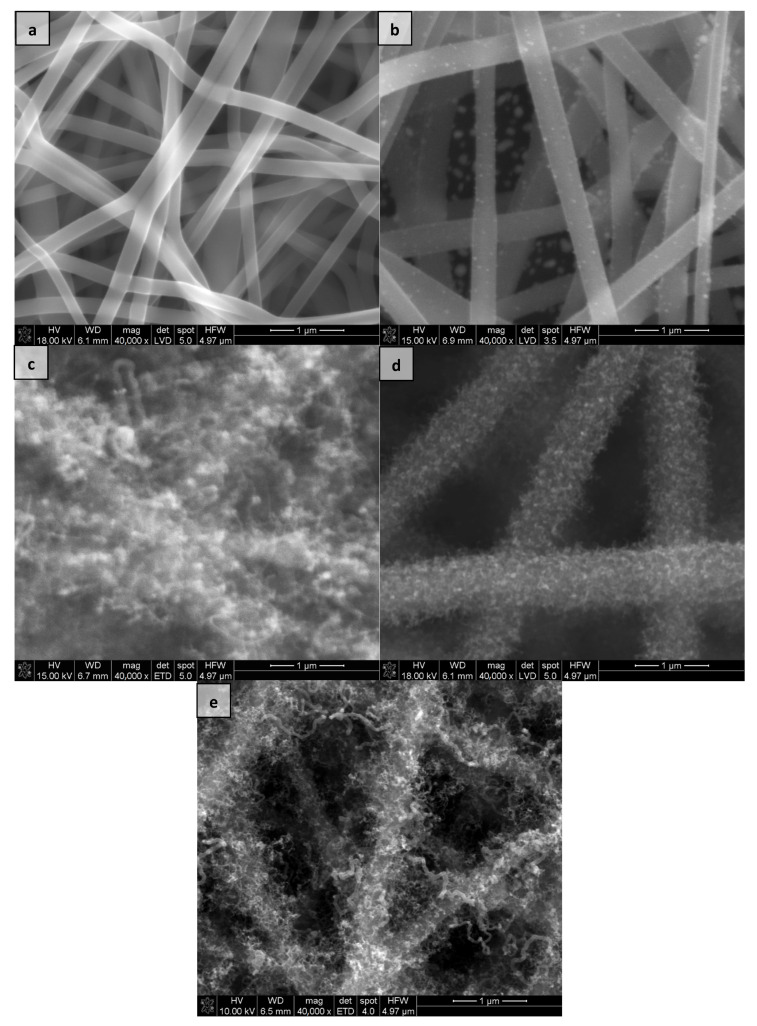
SEM images of (**a**) eCNF, (**b**) eCNF-Co, (**c**) eCNF/CNT[HD]-Co, (**d**) eCNF/CNT[HD]-NiCo, (**e**) eCNF/CNT[LD]-NiCo.

The images obtained from the SEM microscope show the microstructure of the tested materials after synthesis and before dispersion in DMF (Figure 1). All scans were taken with the same magnification–40,000×. 

In the case of pure electrospun carbon nanofibers, the individual nanofibers are clearly visible on the micrograph (Figure 1a). Their diameter is equal to 200–250 nm. Synthesis of this material with the Co nanoparticle precursor causes the incorporation of metal nanoparticles into the nanofibers, clearly visible in the image Figure 1b.

In the case of hierarchical nanocomposites, we can see how carbon nanotubes cover the surface of carbon nanofibers (Figure 1c–e). For the eCNF/CNT[HD]-Co material, the nanotubes that cover the nanofibers seem to be much shorter and less complex than those for other nanocomposites. The total diameter of the nanocomposite is up to 1 µm (Figure 1c).

The eCNF/CNT[HD]-NiCo has the most complex structure. Nanotubes densely cover the surface of nanofibers and the total diameter of this nanocomposite is 1 to 1–1.5 µm (Figure 1d). The total diameter of eCNF/CNT[LD]-NiCo with nanotubes is equal to 0.5–1 µm (Figure 1e).

### 3.2. Wettability

**Figure 2:**
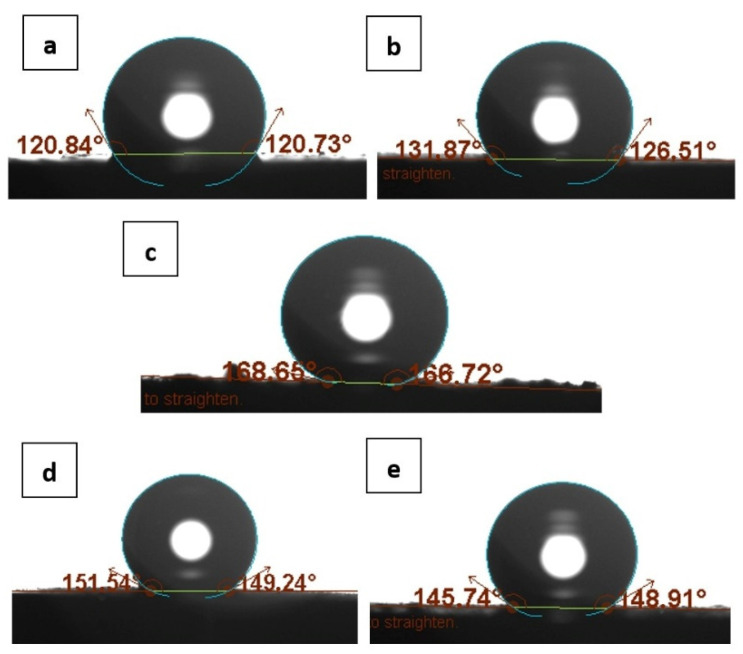
Contact angles of tested layers: (**a**) eCNF, (**b**) eCNF-Co, (**c**) eCNF/CNT[HD]-Co, (**d**) eCNF/CNT[HD]-NiCo, (**e**) eCNF/CNT[LD]-NiCo.

High hydrophobicity is one of the requirements for materials used as intermediate layers. This property of the material allows to eliminate the formation of a water layer between the conductor and the ion-selective membrane [19]. The hydrophobicity of a material is determined by measuring the value of the contact angle in the wettability test. The test involves dropping a drop of water on the material surface that covers the GC disk. Then it determines the contact angle from the photo using One Attension software. Pictures of the determined contact angles are shown in Figure 2.

The average contact angle for carbon fibers was 120.8° which is supposed to be the lowest value obtained, eCNF-Co have a higher contact angle of 129.2°. The highest value of the contact angle, 167.7°, was obtained for the hierarchical nanocomposite eCNF/CNT[HD]-Co. Hierarchical nanocomposites with NiCo particles show a similar contact angle value of 150.4° for eCNF/CNT[HD]-NiCo and 147.3° for eCNF/CNT[LD]-NiCo. These are still a higher value than for eCNF or eCNF-Co.

On the basis of the results, it is easy to see the influence of the nanostructure of the material on its hydrophobicity. Both the addition of nanoparticles and the synthesis of nanotubes visibly improve hydrophobicity. This is due to an increase in the material’s surface/volume ratio. On the basis of the obtained values, it can be expected that the problem of the formation of a water layer in the proposed electrodes will be eliminated. The water layer test is described later in Section 3.6.

### 3.3. Potentiometric Measurements

**Figure 3:**
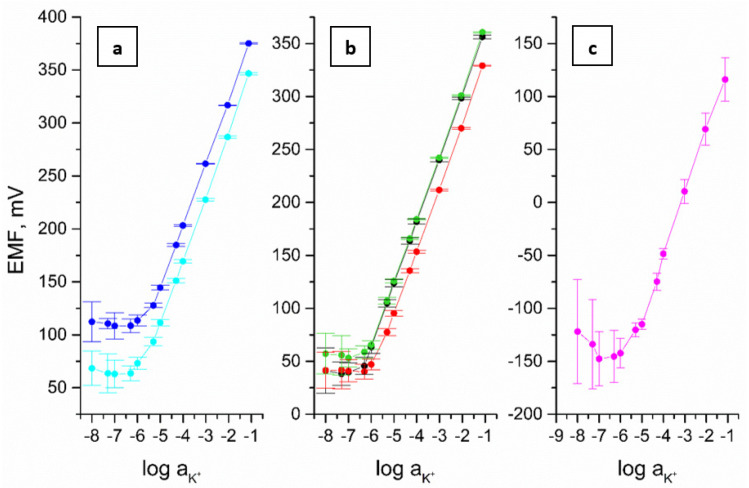
The potentiometric response recorded in KCl solutions for the following electrodes (**a**): GC/eCNF/K^+^-ISM (blue), GC/eCNF-Co/K^+^-ISM (cyan), hierarchical nanocomposites (**b**): GC/eCNF/CNT[HD]-Co/K^+^-ISM (green), GC/eCNF/CNT[HD]-NiCo/K^+^-ISM (black), GC/eCNF/CNT[LD]-NiCo/K^+^-ISM (red) and (**c**): coated disc electrode (magenta) (n = 3).

The dependence of the electromotive force on log $a_{K^{+}}$ determined for the tested electrodes on the basis of triple calibration in solutions with a concentration of 10^−8^–10^−1^ M KCl after 72 h of conditioning in 0.01 M KCl is shown in Figure 3. The calibration curve parameters are summarized in Table 1. The slope determined on their basis is close to the Nerstian value for all tested electrodes.

The detection limit was determined as the activity of K^+^ ions at the intersection of the interpolated linear portions of the calibration curve [20] and collected along with other metrological parameters in Table 1. Obtained values have been compared with the parameters of other described K^+^-selective electrodes using carbon nanomaterials as intermediate layers and valinomycin as ionophore.

The different detection limit values obtained for the subsequent tested electrodes can be explained by the high capacity [23,24,25] and high hydrophobicity [26] of the intermediate layers. The high charge capacity limits the outflow of main ions from the ion-selective membrane. The hydrophobicity prevents the formation of a water layer at the interface. The composition of the water layer may change slowly depending on the composition of the sample and be a source of potential instabilities that deteriorate the detection limit. The poor repeatability of calibration for the coated disc electrode is a result of both the lack of a stable response due to the undefined membrane-substrate interface and the possibility of formation of a water layer. 

The best metrological parameters among tested electrodes were obtained for GC/eCNF/CNT[HD]-NiCo/K^+^-ISM electrode. It is characterised by high reproducibility of normal potential defined by standard deviation. This show that sensors can be used for a long time without repeating of the calibration. Moreover, they have wider linear range and lower limit of detection in comparison to previously reported electrodes [21,22,27,28]. This allows for the determination of lower potassium ion concentrations in real applications.

### 3.4. Stability of the Response

**Figure 4:**
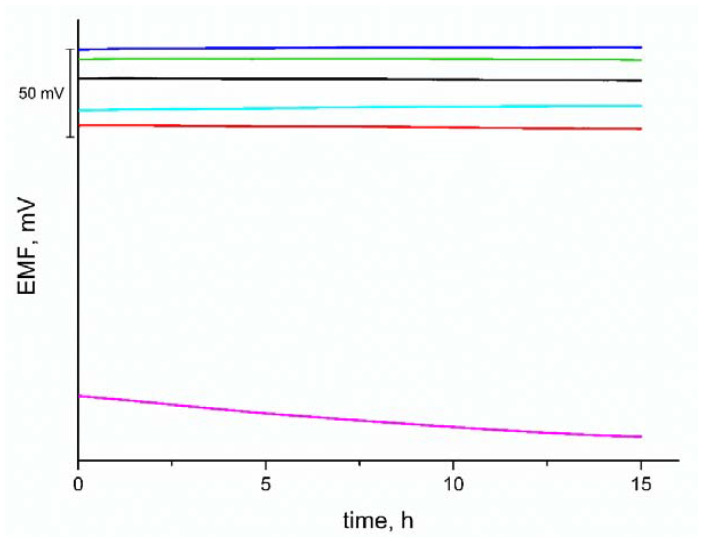
Potentiometric response of the GC/eCNF/K^+^-ISM (blue), GC/eCNF-Co/K^+^-ISM (cyan), GC/eCNF/CNT[HD]-Co/K^+^-ISM (green), GC/eCNF/CNT[HD]-NiCo/K^+^-ISM (black), GC/eCNF/CNT[LD]-NiCo/K^+^-ISM (red) and coated disc electrode (magenta) measured over 20 h after 72 h of conditioning.

Another parameter characterizing ion-selective electrodes is the stability of the potential. This parameter was determined from a 15-h measurement of the potentiometric response in a 0.01 M KCl solution, as shown in Figure 4. The tested electrodes with the intermediate layer show a stable response for the entire duration of the measurement. The response of the coated disc electrode did not reach a stable level during the test. 

The potential drift was calculated to define the long-term stability of the potentiometric response. The drift value is given as the potential/time ratio is equal to 0.09 mV·h^−1^ for GC/eCNF/K^+^-ISM, 0.17 mV·h^−1^ for GC/eCNF-Co/K^+^-ISM, 0.02 mV·h^−1^ for GC/eCNF/CNT[HD]-Co/K^+^-ISM, 0.06 mV·h^−1^ for GC/eCNF/CNT[HD]-NiCo/K^+^-ISM, 0.13 mV·h^−1^ for GC/eCNF/CNT[LD]-NiCo/K^+^-ISM and 1.53 mV·h^−1^ for coated disc electrode. The significantly lower value for solid-contact electrodes is due to the electrochemical properties of the tested materials. The high value of the double-layer capacitance reduces potential drift. The instability of the potential for a coated disc electrode is typical for this type of electrode and is due to the absence of a thermodynamically defined interface between the membrane and the substrate exhibiting different types of conductivity.

### 3.5. Reversibility Test

**Figure 5:**
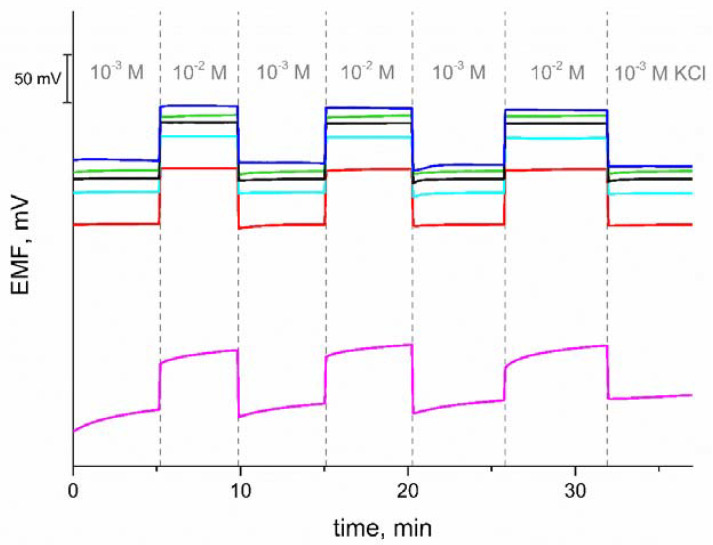
The reversibility test of the GC/eCNF/K^+^-ISM (blue), GC/eCNF-Co/K^+^-ISM (cyan), GC/eCNF/CNT[HD]-Co/K^+^-ISM (green), GC/eCNF/CNT[HD]-NiCo/K^+^-ISM (black), GC/eCNF/CNT[LD]-NiCo/K^+^-ISM (red) and coated disc electrode (magenta) recorded in KCl solutions.

The tested electrodes show good reversibility of the potential response in contrast to the coated disc electrode, as shown in Figure 5. The standard deviation of the potential for 10^−2^ M KCl solutions is equal to 0.8 mV for GC/eCNF/K^+^-ISM, 0.6 mV for GC/eCNF-Co/K^+^-ISM, 0.2 mV for GC/eCNF/CNT[HD]-Co/K^+^-ISM, 0.4 mV for GC/eCNF/CNT[HD]-NiCo/K^+^-ISM, 0.3 mV for GC/eCNF/CNT[LD]-NiCo/K^+^-ISM and 3 mV for coated disc electrode. The electrode response time can also be determined on the basis of rapid changes in the concentration of the solutions. As can be seen for the tested electrodes, after the concentration of the solution is changed, a stable response is obtained after only a few seconds, in contrast to the coated disc electrode, which is characterized by a large potential drift and no stable response.

### 3.6. Water Layer Test

**Figure 6:**
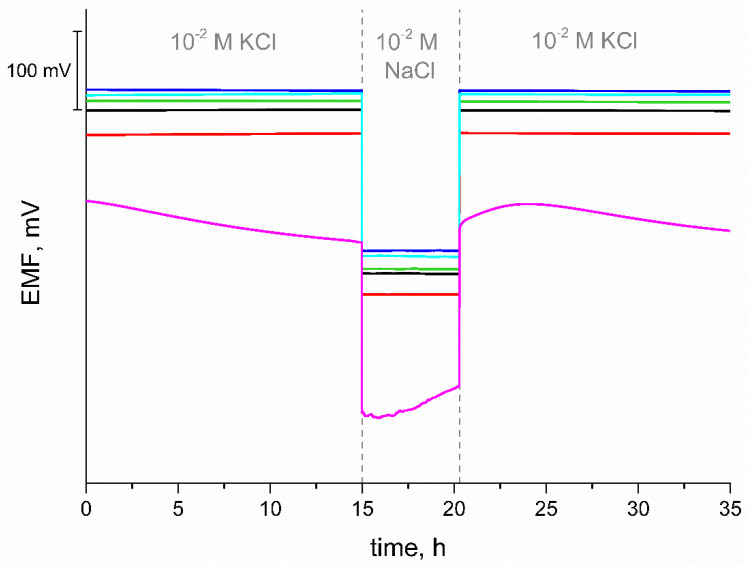
Water layer test of GC/eCNF/K^+^-ISM (blue), GC/eCNF-Co/K^+^-ISM (cyan), GC/eCNF/CNT[HD]-Co/K^+^-ISM (green), GC/eCNF/CNT[HD]-NiCo/K^+^-ISM (black), GC/eCNF/CNT[LD]-NiCo/K^+^-ISM (red) and coated disc electrode (magenta).

The water layer test was performed to check whether an undesirable layer of water has formed during the use of the electrodes. During measurements and electrode conditioning, water penetrates the permeable PVC membrane and can accumulate on the border between the intermediate layer and the ion-selective membrane [29,30]. The formation of the water layer results in deterioration of the potential stability. The method to check whether a water layer has formed has been proposed by Fibbioli et al. [29]. First, the potentiometric response was recorded for about 15 h in 0.01 M KCl, then for 5 h in 0.01 M NaCl solution, and then the interferent solution was changed back to the main ion solution. Based on the results shown in Figure 6, it can be concluded that a layer has formed in the case of the coated disc electrode, as indicated by the characteristic potential drift when the measurement is returned to the main ion solution. For all electrodes with an intermediate layer, no water layer was observed, which is due to the high hydrophobicity of the materials used and allows a stable response of the electrodes, as expected.

### 3.7. Light Sensitivity Test

**Figure 7:**
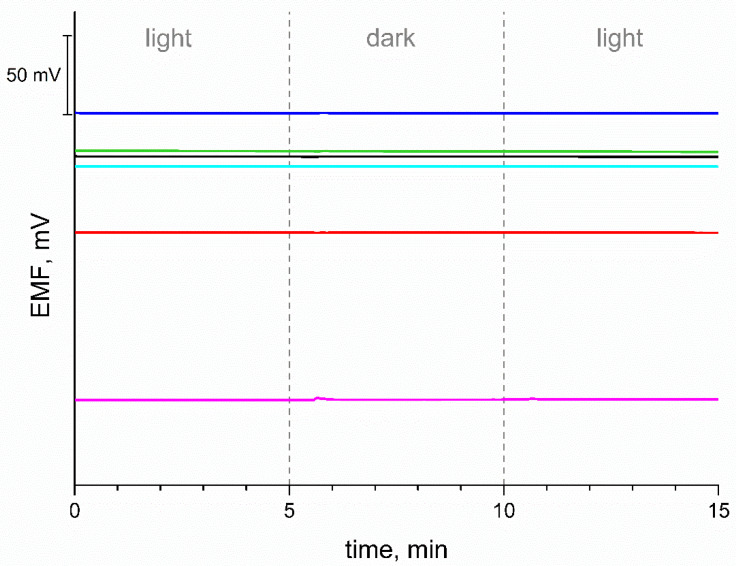
Exemplary potentiometric response during light sensitivity test for GC/eCNF/K^+^-ISM (blue), GC/eCNF-Co/K^+^-ISM (cyan), GC/eCNF/CNT[HD]-Co/K^+^-ISM (green), GC/eCNF/CNT[HD]-NiCo/K^+^-ISM (black), GC/eCNF/CNT[LD]-NiCo/K^+^-ISM (red) and coated disc electrode (magenta).

The influence of light on the potentiometric response of the electrodes was also tested. For this purpose, the potential was recorded in 0.01 M KCl for 5 min in room light, then another 5 min in the dark and again 5 min in room light. In the measurement shown in Figure 7, no significant potential drift was recorded, all tested electrodes did not show sensitivity to light.

### 3.8. pH Sensitivity

**Figure 8:**
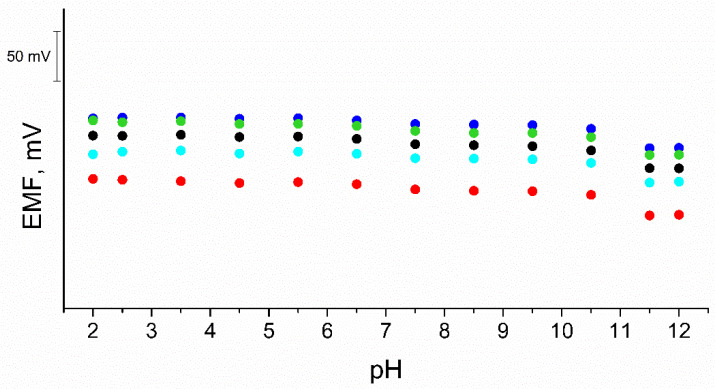
pH sensitivity test of GC/eCNF/K^+^-ISM (blue), GC/eCNF-Co/K^+^-ISM (cyan), GC/eCNF/CNT[HD]-Co/K^+^-ISM (green), GC/eCNF/CNT[HD]-NiCo/K^+^-ISM (black), GC/eCNF/CNT[LD]-NiCo/K^+^-ISM (red).

The examined electrodes were also tested for the effect of pH on the potentiometric response. The test was carried out in 0.01 M KCl solutions with a pH range of 2–12. The pH value was adjusted with NaOH and HCl solutions. The pH range for which the effect of the concentration of hydrogen ions on the potential value has not been recorded is relatively wide and ranges from 2 to 10.5. The deviation in higher pH values may be due to the properties of the ionophore. The recorded potentiometric response is shown in Figure 8.

### 3.9. Chronopotentiometric Measurements

**Figure 9:**
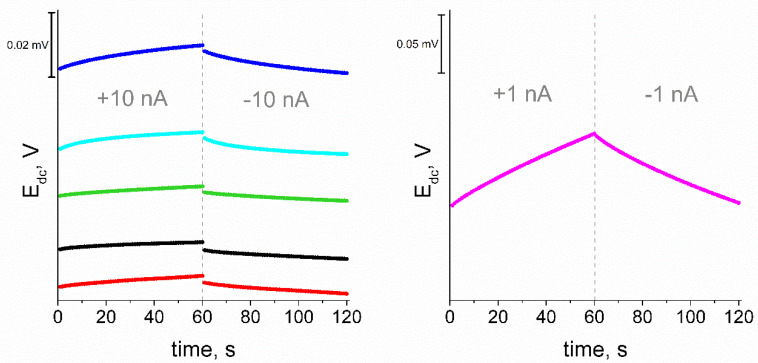
Chronopotentiograms of a GC/eCNF/K^+^-ISM (blue), GC/eCNF-Co/K^+^-ISM (cyan), GC/eCNF/CNT[HD]-Co/K^+^-ISM (green), GC/eCNF/CNT[HD]-NiCo/K^+^-ISM (black), GC/eCNF/CNT[LD]-NiCo/K^+^-ISM (red) and coated disc electrode (magenta).

The electrical parameters of the all-solid-state electrodes were determined using the chronopotentiometry method. The response was recorded at 10 nA flow for the electrodes with intermediate layer or 1 nA for the coated disc electrodes. On the basis of the obtained characteristics, the values of potential drift, resistance and electric capacitance for the tested electrodes were determined. The potential drift and electric capacity were calculated on the basis of line sections of recorded dependences E(t). The parameter values are summarized in Table 2. The obtained chronopotentiograms are presented in Figure 9.

For all electrodes with an intermediate layer, a significant improvement in electrical parameters was observed relative to the control coated disc electrode. There is a visible difference between electrodes with a layer with hierarchical eCNT/CNF nanocomposites and those with materials with only eCNF. The microstructure of the used materials affects the value of electrical capacity. A more complex microstructure means a greater surface area of the material and a higher electric capacity. In order to obtain electrodes with the desired properties, the aim is to lower their resistance and potential drift and increase the value of the electric capacity. As expected, the very deposition of metal nanoparticles on the surface of the eCNF increased its electrical capacity, but much better values were obtained for hierarchical nanocomposites. Moreover, the density of the nanotubes significantly influences the obtained parameters. The highest value was obtained for the GC/eCNF/CNT[HD]-NiCo/K^+^-ISM electrode, but it was characterized by the highest resistance among the electrodes with intermediate layer.

### 3.10. Electrochemical Impedance Spectroscopy Measurements

**Figure 10:**
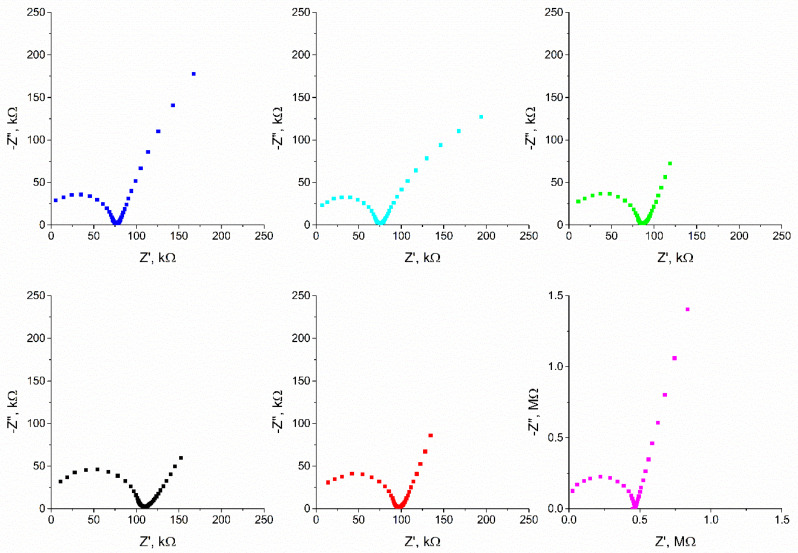
Nyquist plots of a GC/eCNF/K^+^-ISM (blue), GC/eCNF-Co/K^+^-ISM (cyan), GC/eCNF/CNT[HD]-Co/K^+^-ISM (green), GC/eCNF/CNT[HD]-NiCo/K^+^-ISM (black), GC/eCNF/CNT[LD]-NiCo/K^+^-ISM (red) and coated disc electrode (magenta) recorded in 0.01 M KCl.

The electrical properties of the tested electrodes were also examined by electrochemical impedance spectroscopy. The results presented as the Nyquist diagram are shown in Figure 10. The shape of the obtained impedance spectra is characteristic for SC-ISE and CDE. A semicircle can be distinguished in the high and medium frequency regions, and a line in the low frequency region. The semicircle corresponds to the bulk resistance and the geometric capacitance of the ion-selective membrane, while the line corresponds to the characteristics of the processes taking place at the interface between the membrane and the aqueous solution and it allows to determine the electric capacity of the double layer. All of the tested electrodes were covered with the same ion-selective membrane, and the differences obtained between successive groups of electrodes result from the processes taking place in solid contact responsible for ion-electron transduction. The electrical capacity of the tested electrodes was calculated according to the relation presented in the method section, and the obtained values are shown in Table 2. For all tested electrodes, the determined values of the electric charge capacity are lower than those obtained by the chronopotentiometric method, however, the relation between the results is kept well. 

## 4. Application

The operation of the electrodes was tested during the determination of potassium in three tomato juices: fresh juice, commercial juice 1 (content declared by the manufacturer: 200 mg K^+^/100 mL) commercial juice 2. All juices were initially filtered. Three 50 mL samples of each juice were prepared by mixing 1 mL of juice, 5 mL of 1 M NaCl and distilled water. The same concentration of NaCl was also used in the calibration solutions to ensure a constant ionic strength. The determination was performed with the triple addition of the standard solution. The concentration value was determined by the extrapolation method. The results of the determinations made with subsequent electrodes are presented in Table 3.

All electrodes allowed us to obtain similar results in our determinations. Furthermore, the result obtained for Commercial juice 1 is similar to the value declared by the manufacturer.

The standard sample addition method was used to control the quality of the analysis. A clean sample and a sample with the addition of a known amount of standard were examined side by side. Then, the result obtained for the pure sample was subtracted from the result obtained for the sample with the standard addition. The difference was compared to a known amount of standard in the sample and expressed as a percentage. The obtained recovery values indicate that the designed electrodes are useful in chemical analysis. Recovery values were obtained for the analysis of the fresh juice sample. 

## 5. Conclusions

In this work, electrodes with five different intermediate layers were tested: three hierarchical nanocomposites eCNF/CNT: eCNF/CNT[HD]-Co, eCNF/CNT[HD]-NiCo and eCNF/CNT[LD]-NiCo, electrospun carbon nanofibers eCNF and electrospun carbon nanofibers with Co nanoparticles-eCNF-Co as an example of a material formed after the first step of hierarchical nanocomposite synthesis.

The obtained layers were characterized by high hydrophobicity, the highest contact angle was obtained for eCNF/CNT[HD]-Co (168.7°) and slightly lower for eCNF/CNT[HD]-NiCo (150.4°). In addition, electrodes with intermediate layers made of these materials showed the best results. The proposed electrodes differed in the values of the electrical parameters that are closely related to their structure. The highest capacitance was observed for the GC/eCNF/CNT[HD]-NiCo/K^+^-ISM electrode (330±60 µF-CP method) and GC/eCNF/CNT[HD]-Co/K^+^-ISM electrode (260 ± 10 µF-CP method). The lowest detection limit was also obtained for GC/eCNF/CNT[HD]-Co/K^+^-ISM (10^−6.1^ M) and GC/eCNF/CNT[HD]-NiCo/K^+^-ISM (10^−6.3^ M). The most stable response in the long-term stability test and the best reversibility of the response were obtained for GC/eCNF/CNT[HD]-Co/K^+^-ISM 20 µV·h^−1^ and 0.2 mV, respectively. These properties made it possible to obtain useful electrodes, which is confirmed by the results of potassium determinations in juices. 

In conclusion, all proposed materials for intermediate layer were used successfully in potassium selective electrodes. However, their applicability is wider and they can be tested for use in electrodes sensitive to other ions (cations and anions).

## Figures and Tables

**Table 1:** Potentiometric parameters of the investigated K^+^-ISE.

Electrode	Parameter			
	Slope (mV/dec)	E^0^ (mV)	LOD (M)	Linear Range (M)
GC/eCNF/K^+^-ISM	59.45 ± 0.70	435.3 ± 1.7	10^−5.5 ± 0.1^	10^−5^–10^−1^
GC/eCNF-Co/K^+^-ISM	59.32 ± 0.80	401.0 ± 1.7	10^−5.7 ± 0.1^	10^−5^–10^−1^
GC/eCNF/CNT[HD]-Co/K^+^-ISM	59.41 ± 0.54	416.0 ± 1.1	10^−6.1 ± 0.1^	10^−5.3^–10^−1^
GC/eCNF/CNT[HD]-NiCo/K^+^-ISM	59.39 ± 0.80	413.7 ± 0.9	10^−6.3 ± 0.1^	10^−6^–10^−1^
GC/eCNF/CNT[LD]-NiCo/K^+^-ISM	59.40 ± 0.65	385.5 ± 1.1	10^−5.8 ± 0.1^	10^−5^–10^−1^
GC/K^+^-ISM	62.00 ± 4.52	192.3 ± 20.6	10^−5.4 ± 0.2^	10^−5^–10^−1^
C/SWCNT/ K^+^-ISM [19]	57.4	-	10^−6.5^	10^−5^–10^−2^
GC/CRGO/K^+^-ISM [20]	59.2	-	10^−5^	10^−4.5^–10^−2^
CNT paper/K^+^-ISM [21]	56.4	-	7.2 × 10^−6^	10^−5^–10^−1^
GNP/GNP/K^+^-ISM [22]	59	403	-	10^−5.8^–10^−1^

**Table 2:** Electrical parameters of the investigated K^+^- ISE.

Electrode GC/:	R ± SD [kΩ]	ΔE_dc_/Δt ± SD [μV/s]	C ± SD [μF] (CP Method)	C [μF] (EIS Method)
eCNF/K^+^-ISM	91.3 ± 0.7	88.4 ± 1.3	113.1 ± 1.7	89.5
eCNF-Co/K^+^-ISM	93.1 ± 0.1	53.5 ± 0.5	186.8 ± 1.8	125
eCNF/CNT[HD]-Co/K^+^-ISM	94.0 ± 0.8	38.3 ± 1.4	260 ± 10	220
eCNF/CNT[HD]-NiCo/K^+^-ISM	122.4 ± 0.7	31 ± 1.5	330 ± 10	266
eCNF/CNT[LD]-NiCo/K^+^-ISM	105.3 ± 0.2	46.2 ± 1.3	220 ± 10	185
K^+^-ISM	1065 ± 13	1007 ± 70	1.0 ± 0.1	1.18

**Table 3:** Determination of K+ in different samples (SD of 3 measurements).

Electrode	K^+^ Concentration [mg/L]			**Recovery [%]**
	Fresh Juice	Commercial Juice 1	Commercial Juice 2	
GC/eCNF/K^+^-ISM	1980 ± 50	1980 ± 60	2300 ± 70	97
GC/eCNF-Co/K^+^-ISM	2100 ± 50	2030 ± 70	2220 ± 80	102
GC/eCNF/CNT[HD]-Co/K^+^-ISM	1980 ± 40	2090 ± 50	2250 ± 50	101
GC/eCNF/CNT[HD]-NiCo/K^+^-ISM	1940 ± 40	2040 ± 60	2180 ± 70	97
GC/eCNF/CNT[LD]-NiCo/K^+^-ISM	1890 ± 50	2020 ± 60	2150 ± 60	99

## Data Availability

The data presented in this study are available on request from the corresponding author.
